# Diamane-like Films Based on Twisted G/BN Bilayers: DFT Modelling of Atomic Structures and Electronic Properties

**DOI:** 10.3390/nano13050841

**Published:** 2023-02-24

**Authors:** Victor A. Demin, Leonid A. Chernozatonskii

**Affiliations:** Emanuel Institute of Biochemical Physics RAS, 4 Kosygin Street, 119334 Moscow, Russia

**Keywords:** diamane-like material, Moiré structures, DFT modelling, electronic properties

## Abstract

Diamanes are unique 2D carbon materials that can be obtained by the adsorption of light atoms or molecular groups onto the surfaces of bilayer graphene. Modification of the parent bilayers, such as through twisting of the layers and the substitution of one of the layers with BN, leads to drastic changes in the structure and properties of diamane-like materials. Here, we present the results of the DFT modelling of new stable diamane-like films based on twisted Moiré G/BN bilayers. The set of angles at which this structure becomes commensurate was found. We used two commensurate structures with twisted angles of θ = 10.9° and θ = 25.3° with the smallest period as the base for the formation of the diamane-like material. Previous theoretical investigations did not take into account the incommensurability of graphene and boron nitride monolayers when considering diamane-like films. The double-sided hydrogenation or fluorination of Moiré G/BN bilayers and the following interlayer covalent bonding led to the opening of a gap up to 3.1 eV, which was lower than the corresponding values of h-BN and c-BN. The considered G/BN diamane-like films offer great potential in the future for a variety of engineering applications.

## 1. Introduction

Diamane is a fully sp3-hybridized carbon material. It was proposed in 2009 [1] as a hydrogenated AA- or AB-bilayer graphene (G) with interlayer covalent bonds. Changing the hybridization leads to the opening of the bandgap at E_g_~3 eV and an improvement of some mechanical properties [1,2,3,4]. Diamanes can have a wide range of thermal conductivities depending on the adsorbed atoms [5]. The synthesis of genuine diamanes is associated with numerous difficulties [2,6,7]. The first papers detailing the successful synthesis of hydrogenated [8] and fluorinated diamanes [9] were not published until 2020. Earlier attempts led to the formation of diamane-like materials, which are stabilized by external pressure [10], one-side passivation [11] and molecular [12] or covalent bonding with the substrate [13,14].

Diamane-like materials can not only be obtained on a base of AA- and AB-stacked bilayer graphene [15,16]; twisted bilayer graphene [17] can also be used as the basis for another diamane-like material—Moiré diamanes [18,19,20]. The structure of Moiré diamanes is more complicated and contains a large number of non-equivalent interlayer bonds. Their features lead to an increasing of the bandgap and stiffness constant [20,21] as well as to a reduction in thermal conductivity [22] in comparison with untwisted diamanes.

The manipulation of the properties of vertically stacked two-dimensional materials by a twisted angle was named “twistronics” [23]. The twisted angle has been shown to manipulate a wide range of properties, including superconductivity, Moiré excitons, the quantum anomalous Hall effect, the Hofstadter butterfly, both lateral and vertical conductivities, and others [24]. As follows from the review in [25], it is not difficult to prepare such layers with controlled growth and with a high-quality and scalable synthesis, especially with the development of CVD technologies, methods and devices for creating rotated layers with an accuracy of 0.1°. Therefore, we investigated new diamane-like G/BN structures that were designed for applications in nanotwistronics.

One of the parent layers of diamanes can be replaced with a structural analogue of graphene, namely hexagonal boron nitride h-BN. Diamane-like materials based on untwisted G/BN bilayers have been widely investigated theoretically in recent years [26,27]. In the paper [28], modifications of the electronic and structural properties of vertically heterostructured 1–3 layer graphene G and multilayer h-BN on a SiO_x_ substrate induced by high pressure were investigated. Pressure-dependent charge injection experiments on graphene/BN heterostructures have shown that the efficiency of the charge injection decreases with increasing pressure, which indicates a conductor–insulator electronic modification. DFT calculations of twisted graphene/BN with passivation by OH groups from the graphene side, formed using water as the pressure medium, have shown a bandgap opening, which is in agreement with experimental results. These calculations were performed for twisted layers without taking into account the difference in the lattice parameters of graphene and BN.

In the synthesis of diamane-like G/BN structures, it is impossible to obtain a material with an AB diamane structure due to the incommensurability of the layers. Here, we present the results of DFT calculations of a new diamane-like material based on a Moiré G/BN bilayer fully passivated by hydrogen and fluorine atoms, taking into account the lattice mismatch.

## 2. Computational Methodology

The geometric optimization and the calculations of the electronic properties of the considered Moiré G/BN structures were performed by the density functional theory (DFT) method implemented in the VASP package [29,30,31]. We used the generalized gradient approximation with the Perdew–Burke–Ernzerhof (PBE) parametrization of the exchange–correlation functional [32] and the projector augmented wave method [33,34]. The van der Waals interactions were taken into account using the DFT-D3 method [35]. The plane wave energy cutoff was set to 500 eV. The Brillouin zone was sampled using a 2 × 2 × 1 grid for the structures with θ = 10.9° and θ = 25.3°. The distance between the periodically located images was set to be at least 15 Å to avoid the artificial influence of the layers on each other in the non-periodic direction. The tolerance of the SCF convergence was 10^−5^ eV. The atomic structure minimization was carried out until the change in the total energy was less than 10^−3^ eV.

## 3. Results and Discussion

**Figure 1:**
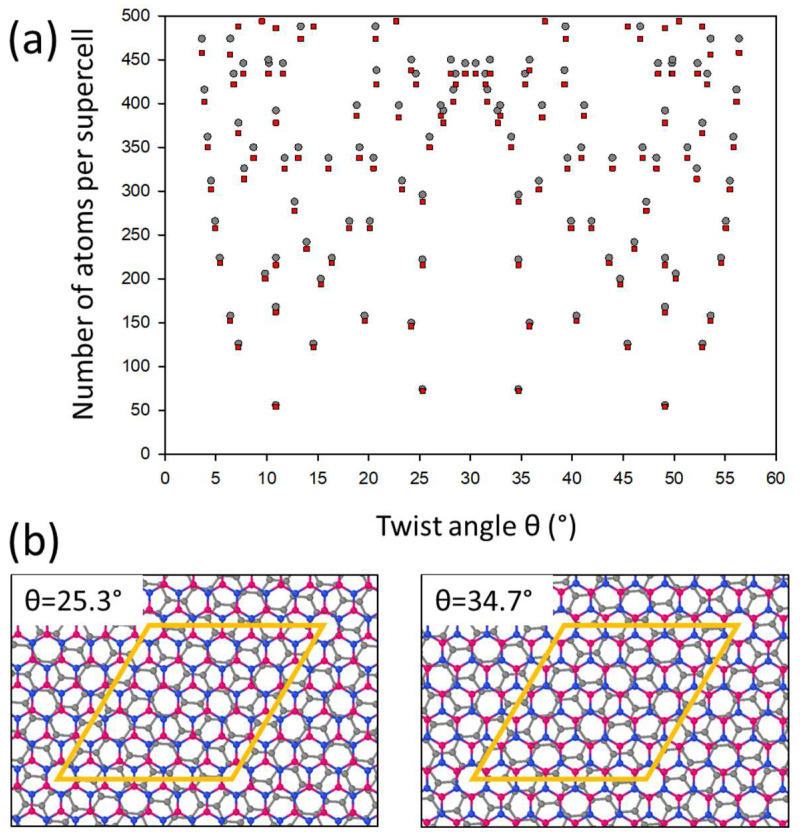
Molecular bonded twisted G/BN bilayers: (**a**) dependence of number of atoms per supercell on twist angle θ (gray circles—number of carbons, red squares—sum of boron and nitrogen atoms); (**b**) top view of the atomic structures with θ = 30° ± 4.7° (gray, red and blue balls denote carbon, boron and nitrogen atoms, respectively).

Graphene and h-BN monolayers are structural analogues and have the same hexagonal lattice. The differences lie in their atomic composition and lattice parameters. When the layers are put onto each other, a mismatch leads to the formation of the Moiré pattern. The lattice mismatch is determined by the formula δ = 1 − a_G_/a_BN_, and common values of δ range between 0.014 and 0.021, corresponding to lattice parameters of a_G_ = 2.46 Å, a_BN_ = 2.49 Å and a_G_ = 2.46 Å, a_BN_ = 2.51 Å, respectively [36]. The two lattices may have different orientations, which are determined by the twisting angle θ. The hexagonal unit supercell of the Moiré pattern with θ = 0° consists of ~9000 atoms for δ = 0.021 and >20,000 atoms for δ = 0.014 [36]. The supercell can be decreased by rotating the graphene layer relative to the BN layer by the twisted angle θ [37]. Our DFT PBE calculations of the isolated monolayers gave a mismatch value of δ = 0.0163. The analysis of the hexagonal Moiré structures with the obtained δ value was carried out to determine the twisted angles θ of the calculated supercells with the smallest number of atoms and the mismatch of the G and BN supercells, where −0.003 < δ < 0.003 (Figure 1a). The smallest supercells corresponded to θ = 10.9° and θ = 25.3° with a stoichiometry of C_56_B_27_N_27_ and C_74_B_36_N_36_ and a mismatch of δ_10_._9_ = −0.0017 and δ_25_._3_ = 0.0028, respectively. The mismatch of these supercells was five- to twelve-times smaller than the mismatch of the G and BN cells. Thus, the layers of these twisted supercells were less compressed or stretched and should be energetically more stable in comparison with untwisted structures [37]. The G/BN structures with angles θ = 30° ± φ, where φ is an arbitrary angle, were the same, but they had different orientations of the BN layer (Figure 1b) in contrast to the identical bilayer graphenes with angles of θ = 30° ± φ. Structures with θ = 30° ± φ will completely coincide when one of them is rotated by 180°, which confirms the identity of the films. The stoichiometry, mismatch and lattice parameters with the formation energies of the twisted G/BN bilayers are given in Table 1.

These hybrid bilayers provided the basis for the diamane-like structures discussed below. Only the structures with their rotation center situated in the middle of the C-atomic hexagon in the carbon layer and coinciding with the center of the atomic BN hexagon of the other layer were considered.

The formation of diamane-like materials is a two-stage process. The adsorption of atoms or molecules onto the outer surfaces of few-layered materials leads to a curvature of layers and changes their hybridization. The inner surfaces become chemically active, which leads to the formation of interlayer bonds. This mechanism has been predicted for untwisted few-layer graphenes [1,38] and few-layer hexagonal boron nitride [39] as well as for twisted bilayers of graphene [18,19,40] and boron nitride [41].

Hydrogen and fluorine atoms are considered as adsorbent. Hydrogens obtained during the hot-filament process were used in the synthesis of diamanes by the Piazza group [6]. In addition, hydrogen or OH groups are formed when water is used as the pressure medium in experiments with few-layered graphenes. Fluorination is more preferable than hydrogenation. The C-F bond is stronger than the C-H bond, and fluorinated diamane is more stable than hydrogenated diamane [9].

**Figure 2:**
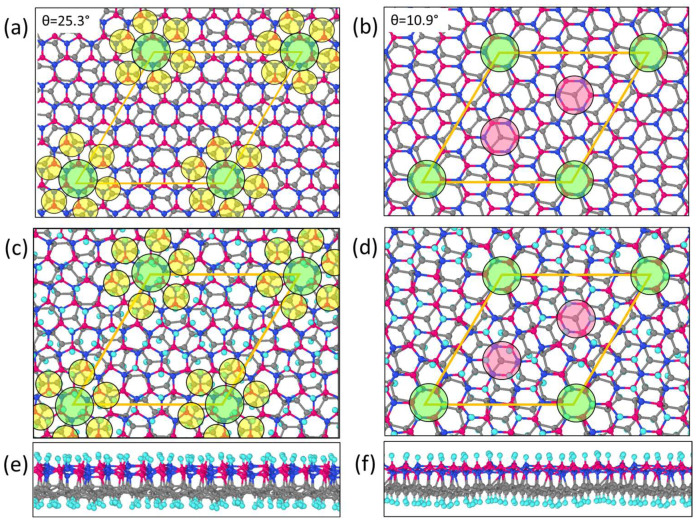
Structural features of twisted G/BN bilayers with θ = 25.3° (**a**) and θ = 10.9° (**b**). Top (**c**,**d**) and side (**e**,**f**) views of the diamane-like structures based on bilayers (**a**,**b**), respectively. C-C and B-N bond “crossings” are marked by yellow circles, AA-stacked areas are marked by green circles and AB-stacked areas are marked by red circles. Structure (**c**) has three interlayer C-N bonds in AA-stacked area, and structure (**d**) has three C-B interlayer bonds.

The chosen hybrid bilayers had structural features, which were important for the adsorption of atoms (Figure 2a,b). The sp^3^ hybrid G/BN bilayers with θ = 25.3° had C–C and B–N bond “crossings”, and the atoms of these bonds did not combine during the process of the formation of the diamane-like material. Not all bond “crossings” are perfect. “Crossings” with a shift of one of the bonds, to which hydrogen adsorption is also preferable, were located in the intermediate regions (Figure 2c,d). This feature is typical for bilayer graphenes with ∼16° < θ ≤ 30° [18]. The G/BN bilayers with θ = 10.9° had two almost AB-stacked areas in the supercell, where three interlayer bonds were formed. The atoms on the bond “crossings” and in the center of the AB-stacked areas had more vibrational freedom in the direction normal to the film surface and, therefore, a higher probability of the formation of bonds with H/F atoms.

The rotational center of the twisted G/BN bilayers was almost in the AA-stacked area, so adsorption occurred, as in the AA-stacked diamane, on alternating atoms (green circles in Figure 2). For this reason, the diamane-like G/BN bilayers with C-N bonds (Figure 2c) and C-B bonds (Figure 2d) in the AA-stacked areas were considered. Further, the closest C-B and C-N pairs were connected, and the rest were functionalized by hydrogen/fluorine atoms. The scheme of adsorption and interlayer connection will depend on many factors such as the pressure, temperature, atmosphere and synthesis time. A high pressure leads to a decrease in the interlayer distance and, therefore, to the formation of more interlayer bonds. A low concentration of H/F in the atmosphere or a short synthesis time will lead to not all the C, B and N atoms being involved in the formation of the interlayer or C/B/N-H/F bonds. This was shown in an example of the fluorination of bilayer graphene [9], where an essentially perfect C_2_F stoichiometry AA- and AB-stacked diamane was reached after only 12 h of fluorination.

In this paper, we considered several possible schemes of fully sp^3^-hybridized structures based on G/BN bilayers with θ = 10.9° and θ = 25.3°. They had C_74_B_36_N_36_H(F)_74_ and C_56_B_27_N_27_H(F)_46_ supercells, respectively. Fewer atoms in the BN layer prevents the formation of the same number of interlayer bonds as in carbon diamanes. Therefore, a sufficiently large amount of adsorbed atoms was chosen compared to non-twisted and twisted carbon diamanes [2] in order to fully obtain the sp^3^ structure.

Structural heterogeneity leads to the formation of non-equivalent interlayer bonds in contrast to untwisted G/BN bilayers [26], which only have C-B or C-N interlayer bonds that are oriented normally to the surface. These diamane-like films with θ = 10.9° and θ = 25.3° had 36 and 32 interlayer bonds, respectively. The structures were obtained and optimized via the DFT PBE method. Optimized structures can be found in the Supplementary Information. Adsorption and interlayer connections changed the hybridization and increased the lattice parameter of the supercells (Table 2). A negative value of the mismatch, δ, for the G/BN bilayer with θ = 10.9° led to a rough graphene surface in contrast to the G/BN bilayer with θ = 25.3° (Figure 2e,f). The surface structure affects the friction properties when using G/BN films in “sandwich” heterostructures [42].

The formation energies were calculated using the formula: E_f_ = (E_tot_ − N_C_E_G_ − N_BN_E_BN_ − N_H2(F2)_E_H2(F2)_)/N, where E_tot_ is the total energy of the G/BN diamane-like structure, N_C_ (N_BN_, N_H2(F2)_) and E_G_(E_BN_, E_H2(F2)_) are the number of carbon atoms (boron + nitrogen and adsorbed atoms) and the energy of the atoms in graphene (B and N in hexagonal boron nitride and adsorbed atom in H_2_ or F_2_ molecules), respectively, and N is the total number of atoms in the calculated cell. The fluorinated structures were more stable than the hydrogenated ones. The negative formation energies demonstrated the higher possibility of their synthesis. The hydrogenated structures had positive energies and could be synthesized at a high external pressure and/or temperature.

The conclusions about the dynamic stability of the considered G/BN diamane-like films was made on the basis of already-published works. The stability of fully carbon diamanes based on hydrogenated/fluorinated AA- and AB-stacked bilayer graphenes was predicted [4] and confirmed by synthesis [8,9]. The changing of one layer to boron nitride keeps the structure stable [26] in both cases with AB_1_ and AB_2_ stacking, where there are only C-B and C-N interlayer bonds, respectively. Untwisted three-layered hybrid C/BN heterostructures are also stable [43]. It is important to note that BNC diamane-like films with the passivation of only the graphene side are stable, which was confirmed by phonon band structure calculations [27]. The influence of the twisting of the parent bilayers on the stability of diamane-like films was also theoretically considered using examples of hydrogenated and fluorinated Moiré carbon [22] and hydrogenated Moiré boron nitride [41] diamanes. So, we concluded that changing one layer of a diamane to BN and twisting the parent bilayers does not lead to the appearance of imaginary frequencies in phonon band structures.

Structural transformations of a G/BN bilayer change its properties. Graphene is a semimetal that has a conduction and a valence band that meet at the Dirac point. Hexagonal boron nitride has a wide bandgap of >6 eV. Dirac cones of graphene are well preserved in combinations of twisted graphene and h-BN [37]. The interaction of the layers leads to the opening of a sufficiently small bandgap of less than 1 meV and to the appearance of dips in the density of the states.

The adsorption of atoms onto graphene and BN distorts their flat surfaces, where atoms leave the plane and bind to hydrogen or fluorine atoms (Figure 2e,f). The surrounding atoms become more chemically active and connect to the adsorbents or to the atoms of the second layer. The π-system of graphene is destroyed, which affects its electronic structure.

**Figure 3:**
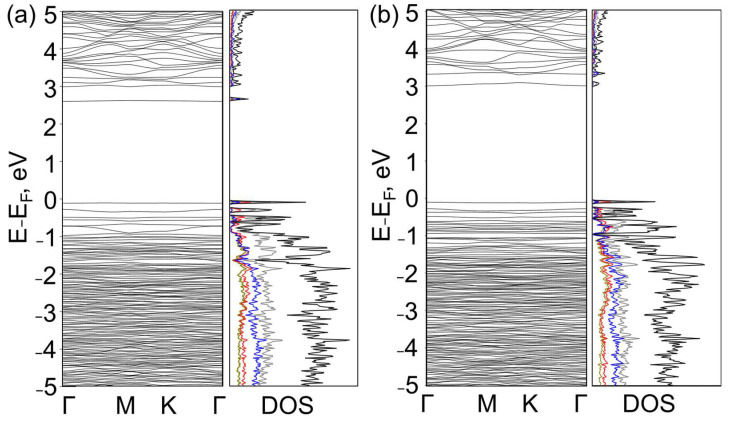
Band structures and densities of states of hydrogenated diamane-like G/BN structures with θ = 25.3° with C-B (**a**) and C-N (**b**) bonds in the AA-stacked area. Cyan, red, blue, gray and black lines correspond to H, B, C, N and total DOS, respectively.

The G/BN structures with θ = 25.3° are semiconductors with a direct bandgap in the Γ-point. The hydrogenated ones had a bandgap of 2.7 and 3.1 eV with C-B and C-N bonds in the AA-stacked area, respectively (Figure 3a,b). The E_g_ values of 2.5 and 3.0 eV corresponded to the fluorinated G/BNs. The twisted heterobilayers had mixed C-N and C-B interlayer bonds in the structure in contrast to the AB-G/BN, where the interlayer bonds were of the same type. Therefore, the difference between the E_g_ of fluorinated twisted structures of 0.5 eV was lower than the 1.3 eV obtained for fluorinated AA-G/BN heterobilayers [26]. The G/BN diamane-like films with θ = 10.9°are also semiconductors with a direct bandgap of up to 3 eV (Table 2). The electronic band structure contains flattened bands near the Fermi level that can lead to unusual features under an electric field, such as in the case of bilayer graphenes with “magic” angles, which have a field-generated Fermi surface with perfect nesting between its holes and electron sheets [44,45].

**Figure 4:**
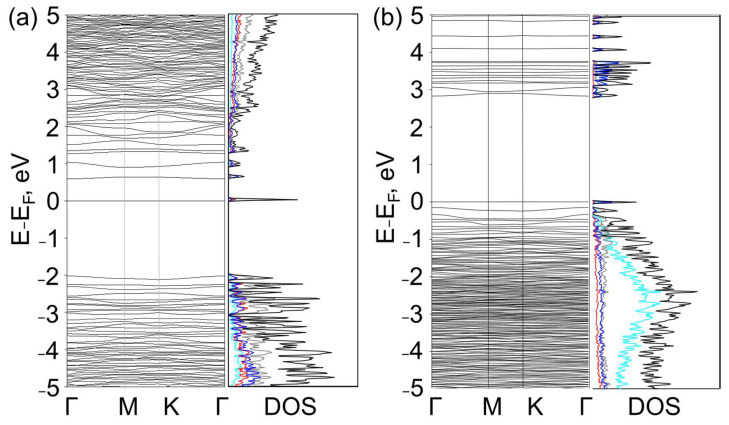
Band structures and densities of states of hydrogenated (**a**) and fluorinated (**b**) diamane-like G/BN structures with θ = 10.9° with C-B bonds in the AA-stacked area. Cyan, yellow, red, blue, gray and black lines correspond to H, F, B, C, N and total DOS, respectively.

To estimate the effect of the quantity of adsorbed atoms on the electronic structure, hybrid G/BN structures with θ = 10.9° and a reduced number of H(F) atoms were calculated. The removal of two atoms on the BN side and one on the graphene side led to a significant change in the properties. The defected G/BN structures C_56_B_27_N_27_H(F)_43_ had almost flat bands crossing the Fermi level (Figure 4). These minizones had widths of 0.0014–0.0674 eV. The view of the band structures showed that the mobility gap, which comprises the highest-occupied and lowest-unoccupied dispersive bands, was large regardless of the coverage. Similar flat bands were observed in the band structures of a diamondol/BN heterostructure [28], where the localizations were induced by a disorder pattern of the interlayer bonds. In the considered cases, the highest value of E_g_ = 3.1 eV corresponded to fluorinated G/BN with C-N bonds in the AA-stacked area, and the lowest E_g_ = 2.6 eV corresponded to hydrogenated G/BN with C-B bonds in the AA-stacked area. Note that Fermi level of the hydrogenated and fluorinated structures lay on the top and bottom of the gap, respectively. This Fermi level shifting of fluorinated structures relative to hydrogenated ones is typical for sp^3^ carbon structures, such as graphanes and fluorographanes. This is a consequence of the negative charges on the fluorine atoms in fluorographene [46]. The reason for the difference in the band structures of the considered twisted G/BN bilayer with various amounts of N_H(F)_ is the density of the adsorbed atoms on the surfaces. The lower density of H(F) makes it impossible to for it to cover all the carbon, boron and nitrogen atoms not involved in the formation of the interlayer bonds. The presence of these sp^2^-hybridizated carbon, boron and nitride species leads to electron localizations and band flattening. Similar hybrid sp^2^/sp^3^ structures based on fluorinated/hydrogenated graphene bilayers with diamane-like domains were investigated earlier [47].

The presence of a Moiré periodic superlattice makes the appearance of flattened zones in the transformed electronic spectrum of graphene in such hybrid structures possible. Moreover, two factors act on its change, namely the covalent bonding of the carbon atoms with the boron and nitrogen atoms, leading to the appearance of wide band gaps in the spectrum, as well as the interaction of the carbon atoms with the inhomogeneously adsorbed hydrogen atoms, leading to the appearance of a narrow miniband, which is similar for the case of a system with hydrogen adsorbed on a diamond surface [48], in particular, and near the Fermi level in our cases. The latter fact is reminiscent of the appearance of a flattened zone with a high electron density in bigraphenes folded at “magic” angles, which is responsible for their superconductivity [49,50]. Therefore, in the twisted G/BN systems considered by us, it became possible to observe similar phenomena. Their study requires special consideration. The presence of a large number of flat bands in the electronic band structures provides the applicability of functionalized Moiré G/BN bilayers as elements of resonant nonlinear optics and optoelectronic devices [41,51].

We believe that double-sided passivated G/BN heterobilayers can be produced by the existing methods. Graphene layers can be grown quite precisely on the surface of boron nitride [52] or rotated by an AFM tip [53]. Further, G and BN layers can be bonded under a low pressure of XeF_2_ as bilayer graphene [9]. Double-sided functionalization can be achieved due to the fact that fluorine atoms can penetrate through the structural defects and edges of a sample. Long-term fluorination leads to the complete coverage of bilayer graphene surfaces and the transition to a diamane-like structure [9]. The covalent interlayer bonding of G and BN under pressure with one-side passivation has already been observed [21]. Thus, the synthesis of the considered hybrid diamane-like materials should be possible in the near future.

Diamane-like films have great potential for application [54,55]. The most-promising applications are next-generation 2D electronic devices. The bandgap and conductivity of these materials can be engineered by varying the twisted angle of the parent bilayers as well as the type and quantity of the adsorbed atoms. The presence of a bandgap allows their use as field-effect transistors and supercapacitors. Functionalized G/BN bilayers should have great mechanical properties due to their diamane-like structures [20,21]. This will allow them to be used as ultrathin insulating and protective coatings or as elements of wearable electronic devices. G/BN diamane-like films are interesting from the point of view of investigating and applying their lubricity properties as three-layered vertical heterostructures of graphene and boron nitride [56]. In particular, the effects of lubricity can be improved for films with fluorine atoms, which have stronger bonds with graphene and boron nitride than hydrogen atoms [9]. The considered structures do not have a center of inversion, so they are piezoelectric materials. The combination of their piezoelectric properties and wide bandgap makes diamane-like G/BN films an excellent candidate for constructing electromechanical devices.

## 4. Conclusions

In summary, a new diamane-like material based on twisted G/BN bilayers was proposed. DFT calculations were performed on the examples of diamane-like films based on parent bilayers with twisted angles of θ = 10.9° and θ = 25.3°. These angles were determined from the obtained dependence on the number of atoms on the twisted angle for bilayers with a lattice mismatch of δ < 1%. The hydrogenation or fluorination of the bilayer surfaces led to interlayer covalent bonding and the formation of a fully sp^3^ hybrid diamane-like structure. The total energy calculations showed the higher stability of the fluorinated structures compared to the hydrogenated ones. Destroying the π-system of graphene led to the bandgap opening up to 3.1 eV. However, the electronic properties of these hybrid diamane-like structures are sensitive to the density and type of the adsorbed atoms, the interlayer connections and the twisting angle of the parent bilayer. A slight decrease in the number of adsorbed atoms led to the appearance of very narrow metal minizones, which were associated with the presence of sp^2^ atoms. The choice of the twisted angle and the density of adsorbed atoms can be used for the precise tuning of the electronic properties of the obtained films. These results indicated that diamane-like hybrid materials could be interesting for applications in next-generation devices.

## Figures and Tables

**Table 1:** Parameters of the considered G/BN supercells.

Unit Cell	Twisted Angle θ, °	Mismatch δ	Lattice Parameter a, Å	Ef, eV
C_56_B_27_N_27_	10.9	−0.0017	13.02	−0.0406
C_74_B_36_N_36_	25.3	0.0028	15.03	−0.0420

**Table 2:** Parameters of considered G/BN diamane-like structures.

Unit Cell	Twisted Angle θ, °	Lattice Parameter a, Å	E_f_, eV/atom	E_g_, eV
Structures with C-B bonds in AA-stacked area				
C_56_B_27_N_27_H_46_	10.9	13.33	0.2025	2.8
C_74_B_36_N_36_H_74_	25.3	15.33	0.2282	2.7
C_56_B_27_N_27_F_46_	10.9	13.48	−0.2624	2.9
C_74_B_36_N_36_F_74_	25.3	15.67	−0.2413	2.5
Structures with C-N bonds in AA-stacked area				
C_56_B_27_N_27_H_46_	10.9	13.33	0.2047	2.5
C_74_B_36_N_36_H_74_	25.3	15.31	0.2395	3.1
C_56_B_27_N_27_F_46_	10.9	13.46	−0.2597	3.0
C_74_B_36_N_36_F_74_	25.3	15.31	−0.2205	3.0

## Data Availability

Data are contained within the article.

## Supplementary Materials

The following supporting information can be downloaded at: https://www.mdpi.com/article/10.3390/nano13050841/s1, File S1.
